# Allopurinol, Benzbromarone, or a Combination in Treating Patients with Gout: Analysis of a Series of Outpatients

**DOI:** 10.1155/2014/263720

**Published:** 2014-02-12

**Authors:** Valderilio Feijó Azevedo, Pedro Grachinski Buiar, Laura Helena Giovanella, Carolina Rossetti Severo, Mauricio Carvalho

**Affiliations:** ^1^Faculty of Medicine, Hospital de Clínicas, Federal University of Paraná, Rua Alvaro Alvin 224, Casa 18, Seminário, 80740260 Curitiba, PR, Brazil; ^2^Pontifícia Universidade Católica do Paraná (PUCPR), Curitiba, PR, Brazil

## Abstract

*Objective*. To profile a sample of gouty patients treated with allopurinol, benzbromarone, or a combination of these two drugs and to describe the impact of this therapy in reducing uric acid levels. *Methods*. An observational, transversal study was performed. We evaluated 48 patients diagnosed with gout who were seen at the Outpatient Rheumatology Clinic of the Federal University of Paraná between January 2009 and November 2010. Clinical data, creatinine serum levels, and basal and posttreatment levels of serum urates, transaminases, and bilirubins were recorded. Uric acid levels were measured in a 24-hour urine sample. Patients were divided into three groups: patients given only allopurinol (A), only benzbromarone (B), and both in combined therapy (A + B). *Results*. The average age of these patients was 56.6 ± 11.4 years, varying from 35 to 81 years. The entire patient group experienced a significant drop in serum urate levels, from 8.5 ± 1.8 mg/dL (0.472 ± 0.1 mmol/L) to 6.7 ± 2.1 mg/dL (0.372 ± 0.116 mmol/L) (*P* < 0.001), regardless of the prescribed medication. The number of patients taking both drugs whose serum uric acid values fell within normal range (men <7 mg/dL (0.38 mmol/L) and women <6 mg/dL (0.33 mmol/L)) was 85.7% (6/7) while this value for the group taking benzbromarone alone was 75% (3/4) and for the group taking allopurinol alone this number was 51.8% (14/27). *Conclusions*. The therapeutic combination of benzbromarone and allopurinol significantly decreased serum urate levels in patients with gout when compared to individual use of each of these agents. This finding is especially important in treating patients who cannot control hyperuricemia with monotherapy. Benzbromarone alone or in combination with allopurinol has an important clinical role in controlling hyperuricemia in patients with gout.

## 1. Introduction

Gout is a form of inflammatory arthritis characterized by the formation of monosodium urate crystals in the joints, synovial liquid, and other tissues [1–4]. It is one of the most prevalent causes of arthritis [4–7] and recent studies suggest that the prevalence and incidence of gout has increased in the last decades [2, 8, 9]. In the USA, the cumulative incidence of gout is 8.6% in men and its prevalence in the general population is approximately 0.5% [1, 2]. The disease affects approximately 1-2% of adult males in occidental countries [1, 4, 7], with a male : female ratio of 3–5 : 1 [5]. According to the Framingham study, 9.2% of men and 0.4% of women had hyperuricemia and 19% of them developed gout [4]. Persistent elevation of serum urate levels is the essential condition leading to the development of gout [1]. About 10% of people with hyperuricemia develop gout and 90% of patients with gout are hyperuricaemic [1]. Risk factors include family history, excessive consumption of alcohol, use of diuretics, kidney disease [1, 2, 6, 9], dietary factors such as elevated consumption of seafood, meat, and vegetables rich in purines [1, 2, 4, 6, 9], elevated body mass index (BMI) [1, 4, 6, 9], age, and gender [1]. Initial clinical presentation is commonly an acute inflammatory monoarthritis affecting the first metatarsophalangeal joint, known as podagra [1, 2, 4]. The presence of urate crystals in the synovial fluid is considered the gold standard in diagnosing gout [1]. The treatment of an acute gout crisis focuses on controlling pain and inflammation [4, 6]. Drugs such as colchicine, anti-inflammatories, and analgesics are part of the therapeutic regime for gout [6]. Once the acute crisis is controlled, drugs that reduce uric acid such as allopurinol are widely prescribed [4, 6].

Benzbromarone is an uricosuric agent that has been used for more than 25 years to control hyperuricemia [10]. It inhibits the main renal urate transporter, URAT-1, an exchanger located in the luminal face of the proximal tubule of the kidney. In doing so, benzbromarone suppresses the reabsorption of uric acid [7, 10]. Benzbromarone is metabolised in the liver by the cytochrome P450 system [11], and as a result should be used with caution in patients with hepatobiliary diseases.

However, benzbromarone is no longer commercially available in USA, and in 2003 it was withdrawn from the European market due to the risk of severe hepatotoxicity [12]. There are case reports of fulminant hepatitis associated with the usage of standard doses of benzbromarone (100 mg/day) during short periods (three months) [13]. Nevertheless, only three cases have been described since the early 1970s and it is debated whether banning benzbromarone was appropriate or if it was done too hastily. In Brazil, sales are permitted by the Brazilian Health Surveillance Agency (ANVISA) and benzbromarone seems to have a safety profile identical to sulfinpyrazone [14]. Besides hepatotoxicity [15], at times of the intense uric acid excretion benzbromarone causes may lead to the formation of uric acid kidney stones [4].

It is recommended to monitor regularly liver function tests—monthly for the first 6 months then every three months. Patients should be advised to stop benzbromarone and seek urgent medical attention if they develop nausea, vomiting, abdominal pain, or jaundice. Benzbromarone also increases the metabolism of warfarin increasing the risk of bleeding in patients using both medications at the same time.

There are few studies in the medical literature evaluating the metabolism of benzbromarone, its side effects, and also its risks and benefits. The objective of this study is to describe the profile of a sample of gouty patients treated with allopurinol, benzbromarone, or a combination of these two drugs, as well as to describe the impact of this therapy on lowering uric acid levels.

## 2. Patients and Methods

### 2.1. Study Population

We evaluated 48 patients diagnosed with gout who were seen at the Outpatient Rheumatology Clinic at the Federal University of Paraná between January 2009 and November 2010. The study was approved by the teaching hospital's ethics committee and informed consent was obtained from all patients.

### 2.2. Procedures

An observational, transversal study was performed. The following data were collected: age; gender; profession; personal history of alcohol, tobacco, or drug use; comorbidities such as obesity, high blood pressure (HBP), diabetes mellitus (DM), hypertriglyceridemia, nephrolithiasis, and osteoarthritis; family history of gout; year of onset (first attack), total number of attacks, average duration of attacks (days), triggering factors, joint affected in the first attack, and other joints involved as the disease evolved; presence or absence of tophi; daily dosage; and how long each medication had been used. Serum creatinine, basal and posttreatment serum levels of uric acid, transaminase, and bilirubin were measured. Uric acid was also measured in a 24-hour urine sample.

### 2.3. Data Analysis

The patients were divided into three groups: patients using only allopurinol (A), only benzbromarone (B), and both in combination (A + B). Treatment for each patient was selected on a case-by-case basis by attending physicians and the head of the clinic. Allopurinol was started in doses from 100 to 300 mg/day, with progressive increases according to therapeutic response. Patients exhibiting hepatic dysfunction were not prescribed benzbromarone. Benzbromarone was started at a dose of 50 mg/day during the first month and was increased later, if necessary, to 100 mg/day. All patients continued to take their usual medications, including colchicine. Uric acid levels were checked every three months. The treatment period with uricosurics ranged from 12 to 18 months. Statistical analysis was performed using SygmaStat 3.5. The results were analysed by variance analysis, *t-*test, paired *t*-test, and nonparametric tests (Wilcoxon and Mann-Whitney), when necessary. The parametric test results are expressed as mean ± standard deviation (SD). A *P* below 0.05 was considered significant.

## 3. Results

Data from the 48 patients studied are summarized in Table 1. The vast majority were males (97.9%). Positive family history of gout was found in more than 15% of patients. The average age of these patients was 56.6 ± 11.4 years, with variation from 35 to 81 years. More than 70% of the individuals had acute monoarthritis in the first metatarsophalangeal joint at some point in the evolution of the disease and approximately 40% of them experienced their first gout attack in this joint. Almost half of the patients had chronic tophaceous gout (Table 1).

Approximately half of the patients identified factors that triggered acute attacks, and the majority of acute attacks were triggered by dietary factors (31.2%) and alcohol consumption (29.1%). Other triggers in our sample included local trauma, cold, physical exercise, and the use of diuretics.

About 80% of the patients had high blood pressure, 20% had diabetes mellitus, 40% had hypertriglyceridemia, and 20% were obese. Cooccurrence of gout and osteoarthritis was identified in approximately 30% of patients. Serum creatinine levels were 1.4 ± 0.8 mg/dL (a variation of 0.6–5.4 mg/dL) and uric acid levels in the urine were 555 ± 356 mg/day (variation of 138–2132 mg/day). 58.3% of the patients were considered to be uric acid hypoexcretors (excretion below 400 mg/day). Approximately 25% of patients experienced nephrolithiasis at some stage of the disease. No patient experienced alteration of bilirubin or transaminases.

**Figure 1:**
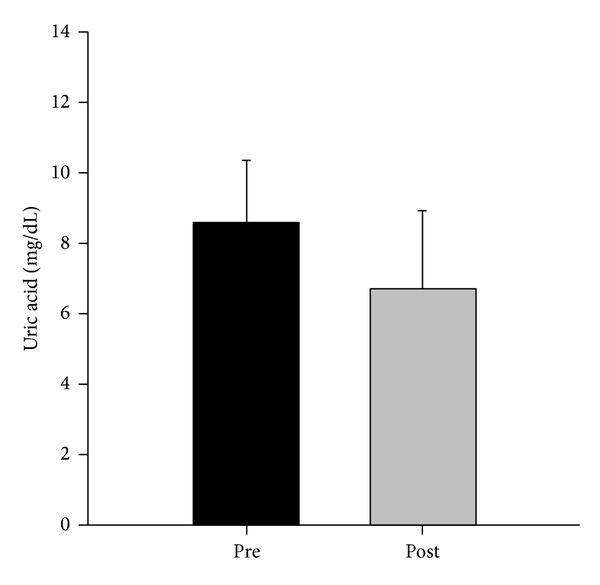
Serum uric acid level pre- and posttreatment (*n* = 48).

**Figure 2:**
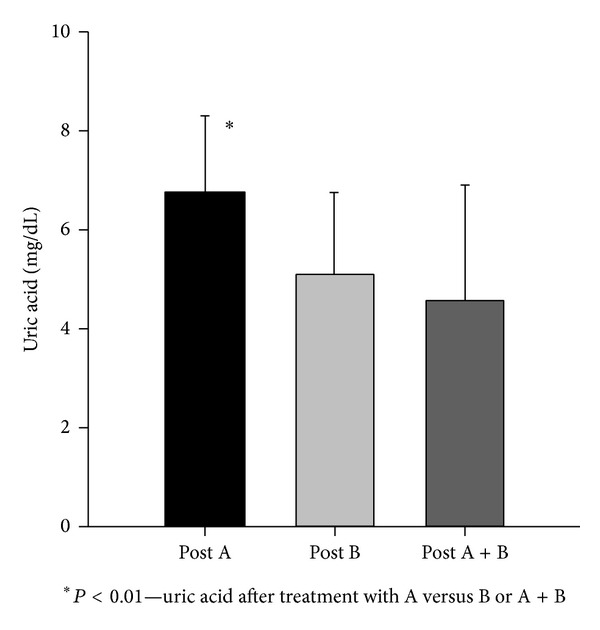
Serum uric acid after treatment with allopurinol (A), benzbromarone (B), or both drugs (A + B).

With regard to the medications used, 31 (64.5%) patients used colchicine, with daily doses between 0.5 and 1 mg. Approximately 40% of the patients were using nonsteroidal anti-inflammatories (NSAIDs) and 18.75% used corticosteroids. The entire patient group experienced a significant drop in uric acid levels, from 8.5 ± 1.8 to 6.7 ± 2.1 mg/dL (*P* < 0.001), regardless of the prescribed medication class (Figure 1). Thirty-eight patients used hypouricemics: allopurinol was prescribed to 27 patients and benzbromarone to four patients, and these two drugs were combined in seven cases. There were no significant differences in age, uric acid levels in urine, or serum urate levels at the beginning of the treatment (Table 2). When comparing the base serum urate levels with the final serum urate levels obtained in this study, there was a significant difference between patients taking only allopurinol and patients taking benzbromarone alone or both drugs together (Table 2 and Figure 2).

The number of patients taking both drugs whose serum urate values were within normal range (men <7 mg/dL and women <6 mg/dL) was 85.7% (6/7), while for the group taking benzbromarone alone this value was 75% (3/4), and in the group taking allopurinol alone it was 51.8% (14/27).

## 4. Discussion

At a time when new synthetic and biological medications to treat gout are being considered, we revisited a molecule that is already known and used in the therapeutic control of this ancient disease. Our study analysed 48 gouty patients followed in a specialised outpatient rheumatology clinic. Eighty percent (38/48) received hypouricemics. In most cases, allopurinol was the drug of choice to reduce hyperuricemia (27/38, 71%). All drugs were selected after a complete patient evaluation by the attending physician.

We observed that, in patients taking allopurinol alone, serum urate levels were higher than in those taking benzbromarone alone or taking a combination of both drugs (Table 2).

There is evidence that standard doses of benzbromarone (100 mg/day) produce stronger hypouricemic effects than standard doses of allopurinol (300 mg/day) [11, 15, 16]. In two series, benzbromarone reduced uric acid concentration by 54% to 63% and uric acid levels were inferior to 5 mg/dL [15, 17].

In a study conducted by Perez-Ruiz et al., 53% of patients who received allopurinol and 100% of patients who received benzbromarone achieved plasma urate levels considered optimal for disease control [15]. In another study in which only patients intolerant to allopurinol were analysed, 92% of patients who received benzbromarone were successfully treated, maintaining serum urate levels at around 5 mg/dL [4, 11, 17]. In our study, allopurinol brought serum urate levels to within normal range in 51.8% of the patients, benzbromarone alone reduced urates to normal levels in 75% of patients, and combination therapy normalized uricaemia in 85.7% of patients.

In a recent publication Reinders et al. [18] compared efficacy and tolerability between allopurinol 300–600 mg/day and benzbromarone 100–200 mg/day. When doses considered to be lower than the standard were used, uric acid levels were brought to normal levels in 26% of cases using allopurinol and 52% of cases using benzbromarone. With higher doses, the success rate was similar: 78% for both drugs.

Benzbromarone can be considered for patients with low urate excretion, except if there is a previous history of nephrolithiasis [15]. Patients intolerant to allopurinol or who experience unsatisfactory reduction of urate levels or even primary hyperuricemia with deficiency in kidney urate excretion (<400 mg/24 h) but normal kidney function can be treated with benzbromarone [4]. In our study, the majority of patients using benzbromarone were hypoexcretors of uric acid.

Benzbromarone has limited effectiveness in patients with kidney insufficiency [19] but can be effective in patients with moderate kidney insufficiency [11]. Although this drug is less effective in patients with lower creatinine clearance, an important reduction in uric acid levels was observed in these patients [15].

The serum creatinine levels of patients using benzbromarone varied greatly, with values between 0.8 and 2.3 mg/dL. We did not measure the patients' 24-hour creatinine clearance.

In the patient group taking both allopurinol and benzbromarone simultaneously, initial serum urate levels of 8.2 ± 1.4 mg/dL decreased about 47.25% to 4.6 ± 2.3 (*P* < 0.001) after one year. This result can be considered superior when compared with the patient group taking allopurinol alone (Figure 2) and similar to the result obtained from the group taking benzbromarone alone. In a study performed by Yamamoto and collaborators, an interaction between allopurinol and benzbromarone was analysed. The authors concluded that the interaction did not affect the plasmatic concentration of oxipurinol (the main allopurinol metabolite) but caused an increase in oxipurinol clearance [20]. Oxipurinol levels were not measured in our patients.

According to Perez-Ruiz et al., benzbromarone, used alone or combined with allopurinol, is superior to therapy with allopurinol in reducing gouty tophi [21]. Our study did not evaluate the impact of benzbromarone on this clinical outcome.

This study has limitations: first of all, the choice of therapy was not randomized but was chosen by the attending physician according to each patient's profile. Similarly, patient followup was not blind; that is, doctors and patients were aware of which medication was being used in each case. Moreover, although 11 patients received benzbromarone, the number of patients taking this drug alone (*n* = 4) was very small.

However, we believe that benzbromarone, alone or in combination with allopurinol, has an important clinical role in controlling uricaemia of patients with gout. Patients should be selected carefully to take benzbromarone, and those with liver disease or advanced chronic kidney disease should be excluded. Of course, a prospective, randomized double-blind clinical trial involving multiple centres is still needed.

As stated by Lee and collaborators in a recent revision [22], “availability of benzbromarone to treat selected cases of gout would be especially interesting, particularly to patients in whom allopurinol produces insufficient response or toxicity.”

## 5. Conclusion

The therapeutic combination of benzbromarone and allopurinol significantly decreased serum urate levels in patients with gout when compared to the individual use of each of these agents. This finding is especially important in treating patients who cannot control hyperuricemia with monotherapy.

The lack of serious adverse effects associated with benzbromarone in our study is probably related to the selection of suitable patients. We believe that benzbromarone plays an important role in controlling hyperuricemia and clinical remission of patients with refractory gout.

## Figures and Tables

**Table 1:** Basal characteristics of the study population (*n* = 48).

	(%)
Male gender	47 (97.9%)
Age, years (mean ± SD)	56.6 ± 11.4
Alcohol abuse	18 (37.5%)
Smoking	4 (8.3%)
Hypertension (BP ≥ 140/90 mmHg)	38 (79.1%)
Diabetes mellitus (fasting glucose > 126 mg/dL)	10 (21%)
Hypertriglyceridemia ( ≥150 mg/dL)	20 (41.6%)
Obesity (BMI ≥ 30)	9 (18.75%)
Osteoarthritis	16 (33.3%)
Positive family history	8 (16.7%)
Precipitating factor	23 (48%)
Podagra	35 (73%)
Chronic tophaceous gout	23 (48%)

**Table 2:** Change in serum uric acid (UA) with allopurinol, benzbromarone, or both drugs.

Parameter	Allopurinol	Benzbromarone	Both drugs	*P*
Age (years)	58.5 ± 10	45.7 ± 8.3	57.8 ± 11.8	0.093
Urine UA (mg/day)	575 ± 397	595.5 ± 466	461.8 ± 251.5	0.857
Pretreatment serum UA (mg/dL)	9 ± 1.6	9.1 ± 1	8.2 ± 1.4	0.499
Posttreatment serum UA (mg/dL)	6.7 ± 1.5	5.1 ± 1.6	4.6 ± 2.3	0.01*

*Serum UA posttreatment with allopurinol versus serum UA posttreatment with benzbromarone or both drugs.
